# Short range biaxial strain relief mechanism within epitaxially grown BiFeO_3_

**DOI:** 10.1038/s41598-019-42998-x

**Published:** 2019-04-30

**Authors:** In-Tae Bae, Shintaro Yasui, Tomohiro Ichinose, Mitsuru Itoh, Takahisa Shiraishi, Takanori Kiguchi, Hiroshi Naganuma

**Affiliations:** 1 Small Scale Systems Integration and Packaging Center, State University of New York at Binghamton, Binghamton, New York, 13902 USA; 2 Department of Physics, State University of New York at Binghamton, Binghamton, New York, 13902 USA; 3 Laboratory for Materials and Structures, Tokyo Institute of Technology, 4259-J2-19, Nagatsuta-cho, Midori-ku, Yokohama 226-8503 Japan; 4 Department of Applied Physics, Graduate School of Engineering, Tohoku University, Sendai, 980-8579 Japan; 5 Institute for Materials and Research, Tohoku University, Sendai, Japan

**Keywords:** Ferroelectrics and multiferroics, Surfaces, interfaces and thin films

## Abstract

Lattice mismatch-induced biaxial strain effect on the crystal structure and growth mechanism is investigated for the BiFeO_3_ films grown on La_0.6_Sr_0.4_MnO_3_/SrTiO_3_ and YAlO_3_ substrates. Nano-beam electron diffraction, structure factor calculation and x-ray reciprocal space mapping unambiguously confirm that the crystal structure within both of the BiFeO_3_ thin films is rhombohedral by showing the rhombohedral signature Bragg’s reflections. Further investigation with atomic resolution scanning transmission electron microscopy reveals that while the ~1.0% of the lattice mismatch found in the BiFeO_3_ grown on La_0.6_Sr_0.4_MnO_3_/SrTiO_3_ is exerted as biaxial in-plane compressive strain with atomistically coherent interface, the ~6.8% of the lattice mismatch found in the BiFeO_3_ grown on YAlO_3_ is relaxed at the interface by introducing dislocations. The present result demonstrates the importance of: (1) identification of the epitaxial relationship between BFO and its substrate material to quantitatively evaluate the amount of the lattice strain within BFO film and (2) the atomistically coherent BFO/substrate interface for the lattice mismatch to exert the lattice strain.

## Introduction

BiFeO_3_ (BFO) has been known for its multiferroic property, i.e., ferroelectricity and *G*-type antiferromagnetism showing up well above room temperature^1,2^. This material had drawn little attention because its spontaneous polarization value was originally measured at merely ~6 μCcm^−2^, which is too weak for practical device application^1,3^. However, the availability of high quality bulk BFO materials revealed its true spontaneous polarization value of ~100 μCcm^-2^ in the early 2000s^4,5^. This has triggered extensive research efforts on BFO due to the significant implication in spintronics and smart energy, i.e., low-energy consumption, applications^6^. In particular, BFO thin films have been grown epitaxially on a wide variety of single crystal oxide substrates in an attempt to modify its physical properties by making use of the biaxial lattice strain induced by the lattice mismatch with the substrate materials. Since the physical properties of the BFO films are tightly bound to their atomistic structural details, a number of studies have attempted to investigate the details about crystal structural modification within BFO thin films by using a variety of substrate materials that imparts different amounts of the lattice misfit. As a result, the BFO thin films have been reported to grow as a number of crystal structures such as, tetragonal-like^7–11^, orthorhombic^12^, monoclinic^13–15^, orthorhombic-like monoclinic^16^, and triclinic^17^ in addition to its equilibrium crystal structure of rhombohedral, i.e., space group (SG) of *R*3*c*^3,18–21^. Despite these efforts, the structural details within the BFO thin films and their impact on the physical properties such as spontaneous polarization and antiferromagnetism are continuously debated primarily owing to its remarkably complex nature as pointed out by recent reviews^22,23^. In order to address this challenging issue, we have recently proposed a novel methodology in which the two most effective structural analysis techniques of x-ray diffraction (XRD) and transmission electron microscopy (TEM) are utilized together in a complementing manner as follows^21,23^. Since TEM technique can provide wide range reciprocal space information, it readily reveals crystal symmetry information by revealing the geometries among reciprocal lattices along multiple crystal orientations. As a result, the crystal symmetry can be unambiguously identified when the geometries among reciprocal lattices match the structure factor calculation results. Once the overall crystal symmetry is clearly identified, the small variations, i.e., the minute distortions caused by the lattice misfit with the substrate within the identified crystal symmetry, can be further investigated with XRD technique that focuses on highly localized area in reciprocal space with exceptional precision.

It should be noted that the hexagonal notation rather than the pseudocubic one is highly recommended to use to accurately describe structural details of the rhombohedral, i.e., SG of *R3c*, BFO^21,23,24^. This is because pseudocubic notation disregards the ~0.55° of rhombohedral distortion in BFO unit cell and *subsequent rhombohedral shifts* in basis atom locations. As a result, the pseudocubic notation cannot interpret some of Bragg’s reflections that are specifically related to the rhombohedral characteristic as demonstrated in recent reports^21,23,24^. Thus, hereafter, *hexagonal notation* is used to accurately describe rhombohedral BFO in this work unless otherwise mentioned. Another parameter to quantitatively evaluate the biaxial strain exerted on BFO films is the misfit lattice strain with substrate materials. Conventionally, the misfit lattice strains are estimated by assuming BFO and the substrate materials as pseudocubic crystals^25^. In this assumption, the growth orientation of BFO film is simply assumed to be the same as that of the substrate material. While this could be reasonable with some substrate materials that have similar lattice parameters as BFO, the possibility of BFO growth having different crystal orientations is excluded. Thus, this assumption is not expected to accurately estimate the misfit lattice strain if a BFO film grows on a substrate having a different crystal orientation from that of the substrate as pointed out previously^21,23^.

In this study, the crystal structures and growth mechanisms of the epitaxial BFO films grown on La_0.6_Sr_0.4_MnO_3_(LSMO)/SrTiO_3_(STO) and YAlO_3_ (YAO) substrates are studied using TEM and XRD techniques. The result clearly demonstrates:

1. The importance of the direct observation on the BFO/substrate interface to determine either the biaxial strain evaluated by the lattice mismatch is exerted toward the BFO film with atomistically coherent lattice plane, or it is relaxed by introducing lattice imperfections such as dislocations.
2. BFO film grows retaining the *rhombohedral symmetry* despite a large lattice mismatch of ~6.8% with its substrate, but with an unusual epitaxial relationship. This result is important in that it answers the question that “*Does BFO grow another metastable phase if a substrate imparting larger compressive strain than LaAlO*_*3*_ *is used?*”^9,11,16^.
3. The identification of the epitaxial relationship between the BFO film and the substrate material should precede to ensure accurate evaluation of the lattice mismatch.

## Results and Discussion

**Figure 1:**
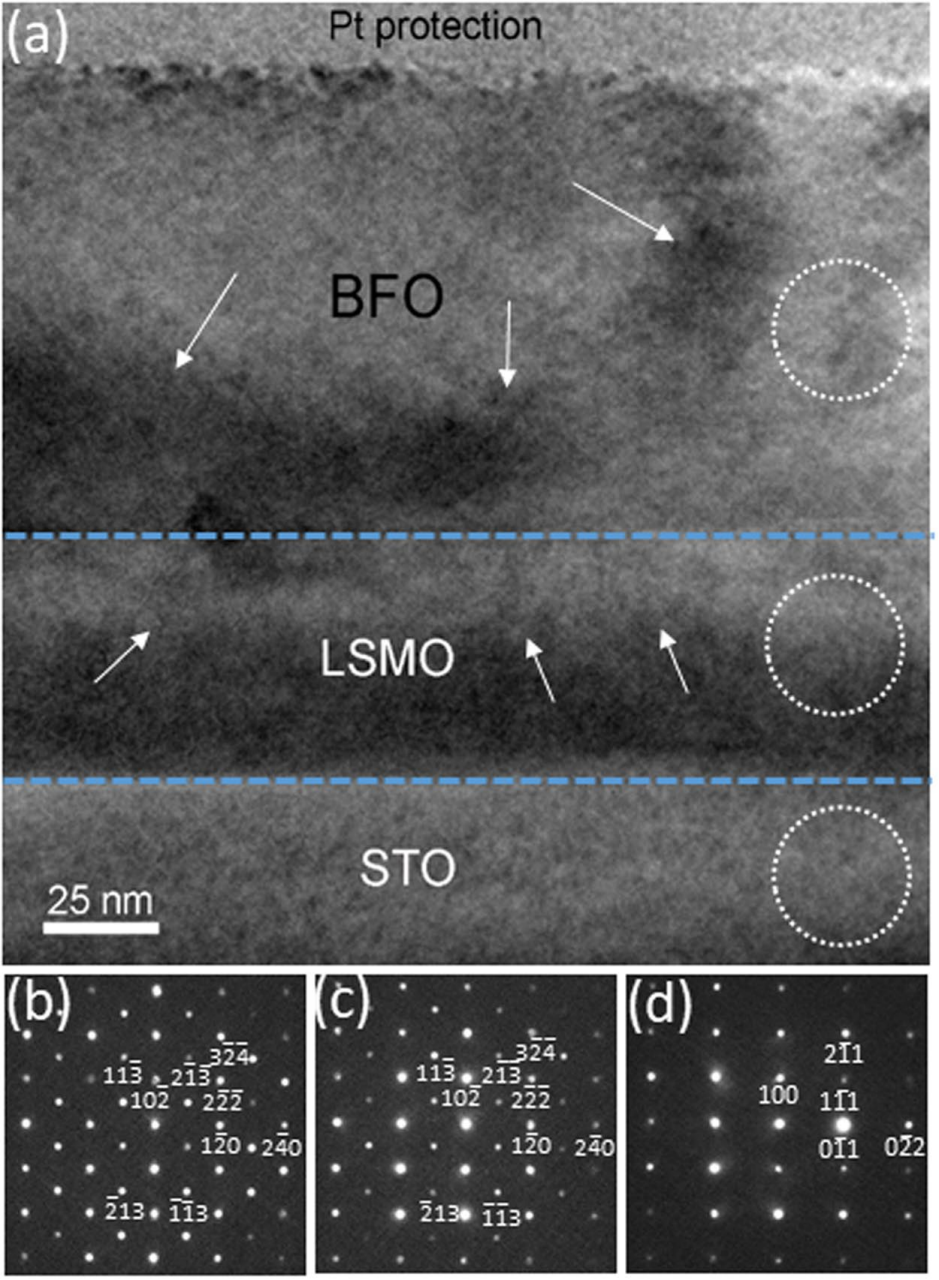
(**a**) A cross-sectional BF TEM image of BFO layer grown on LSMO buffered STO substrate along [011]_STO_ zone axis. The strain-induced contrasts are denoted with arrows. The NBED patterns from BFO, LSMO, and STO are shown as (**b**–**d**), respectively.

Figure 1(a) is a cross-sectional bright-field (BF) TEM image of the BFO/LSMO/STO sample along [011]_STO_ orientation, which shows overall microstructural characteristics. Note that [011]_STO_ zone axis is chosen because this zone axis is proven to reveal the subtle symmetry difference between rhombohedral, i.e., space group of *R3c*, and cubicperovskite, space group of $Pm\bar{3}m$, within BFO films grown on cubicperovskite substrates previously^16,19–21,23^. A BFO layer of ~95 nm is confirmed to grow on a ~50 nm LSMO electrode layer grown directly on the STO substrate. Note that both of the BFO and LSMO layers show stress/strain contrasts as denoted by white arrows, whereas the STO substrate show no such contrast. This implies both of the BFO and LSMO layers could be under the lattice strains caused by the lattice mismatch with STO substrate. In order to investigate the crystal structures of LSMO and BFO, nano-beam electron diffraction (NBED) technique was applied to BFO, LSMO and STO with a probe size of ~40 nm as denoted by the three circles in each material. The corresponding NBED patterns are shown in Fig. 1(b–d), respectively. It should be noted that while the symmetry of the pattern in Fig. 1(d), i.e., STO, matches that of [011] zone axis of cubicperovskite, those in Fig. 1(b), i.e., BFO, and 1(c), i.e., LSMO, correspond to [211] zone axes of rhombohedral with the rhombohedral signature Bragg’s reflections such as $2\overline{13}$, $11\bar{3}$, $\bar{2}13$ and $\overline{11}3$. Note that the indices for BFO are based on *hexagonal notation* as mentioned earlier. These reflections have been confirmed to be used as the fingerprints of rhombohedral symmetry within BFO because they show up in rhombohedral BFO only^16,19–21,23^. In other words, they overlap none of Bragg’s reflections from the other BFO symmetries (including the pseudocubic) as discussed previously^23^. The epitaxial relationships found among BFO, LSMO, and STO are as follows:

1 $${[211]}_{{\rm{BFO}}}//{[211]}_{{\rm{LSMO}}};{(10\bar{2})}_{{\rm{BFO}}}//{(10\bar{2})}_{{\rm{LSMO}}}$$

2 $${[211]}_{{\rm{LSMO}}}//{[011]}_{{\rm{STO}}};{(10\bar{2})}_{{\rm{LSMO}}}//{(100)}_{{\rm{STO}}}$$

**Figure 2:**
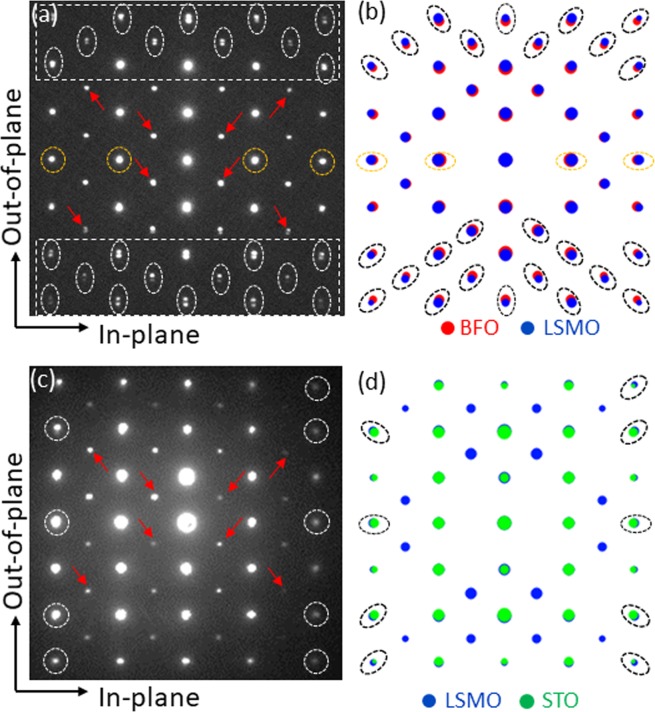
NBED patterns from the BFO/LSMO (**a**) and LSMO/STO (**c**) interfaces. The corresponding structure factor calculation results by assuming no lattice strains at the BFO/LSMO and LSMO/STO interfaces are shown (**b**) and (**d**), respectively. Note that the Bragg’s reflections denoted by red arrow in (**a**) and (**c**) are resulting from double diffraction.

In order to acquire a direct insight about the status of the lattice mismatches among the three materials, the NBED patterns were acquired from the BFO/LSMO and LSMO/STO interfaces as shown in Fig. 2(a,c) respectively. Note that the high index Bragg’s reflections from BFO and LSMO (encircled in white) split along out-of-plane orientation in Fig. 2(a). The rectangle areas at the top and bottom are enlarged in supplementary Fig. S1 to show the split clearly. Besides, the in-plane Bragg’s reflections from BFO and LSMO (encircled in orange) perfectly overlap with no split, indicating that the in-plane lattice spacing of BFO is forced to match that of LSMO. This strongly suggest that the BFO/LSMO interface is atomistically *coherent* along in-plane orientation. In other words, the lattice mismatch between BFO and LSMO exerts the lattice strain in the BFO film by forcing the in-plane lattice spacing of BFO, i.e., ${(1\bar{2}0)}_{{\rm{BFO}}}$, to match that of LSMO, i.e., ${(1\bar{2}0)}_{{\rm{LSMO}}}$. In order to visualize how the NBED pattern in Fig. 2(a) is different when *no lattice strain* exists in the BFO film, the structure factor, *F*_*hkl*_,

$${F}_{hkl}=\sum _{n}{f}_{n}\,\exp [2\pi i(h{x}_{n}+k{y}_{n}+l{z}_{n})],$$

where *hkl* represents a specific Bragg’s reflection; *f*_*n*_ is the atomic scattering factor for atom *n* at fractional coordinates (*x*_*n*_, *y*_*n*_, *z*_*n*_), was calculated by using the crystallographic data, i.e., space group, lattice parameter, and basis atom locations, of *unstrained* rhombohedral BFO^26^ and *unstrained* rhombohedral LSMO^27^ materials in tandem with epitaxial relationship (1) as shown in Fig. 2(b). Note that the high index Bragg’s reflections from BFO and LSMO (encircled in black) split radially with respect to the direct beam located at the center. This is in clear contrast with the splits showing up along only out-of-plane orientation in Fig. 2(a). In addition, the in-plane Bragg’s reflections from BFO and LSMO (encircled in orange) in Fig. 2(b) split slightly along in-plane orientation owing to the different in-plane lattice spacings, i.e., *d*_in-plane_ of BFO = 0.2787 nm^26^ and *d*_in-plane_ of LSMO = 0.2742 nm^27^, whereas the corresponding Bragg’s reflections in Fig. 2(a) (encircled in orange) show no sign of splits. Thus, Fig. 2(a,b) clearly demonstrate the difference of the NBED patterns between with *strain*, i.e., Fig. 2(a), and with *no strain*, i.e., Fig. 2(b), within the BFO film. Note that the reflections denoted with red arrows in Fig. 2(a,c) are the result of double diffraction^28,29^. Now let us turn our attention to the underlying LSMO/STO interface. An NBED pattern from the interface is shown in Fig. 2(c). It is readily noticed that the Bragg’s reflections from LSMO and STO overlap completely with no sign of splits. This result is similar to the structure factor calculation for the unstrained LSMO/STO interface that uses the crystallographic data of unstrained LSMO^27^ and *unstrained* STO^30^ together with epitaxial relationship (2) [see Fig. 2(d)]. However, it is worth noting that while most of the Bragg’s reflections from LSMO and STO overlap in Fig. 2(d), some of high index Bragg’s reflections encircled in black split up slightly due to the minute lattice spacing differences along in-plane and out-of-plane orientations between LSMO and STO. On the other hand, the corresponding Bragg’s reflections encircled in white in Fig. 2(c) show no sign of the splits. This indicates that the in-plane lattice spacing of LSMO is forced to match that of STO.

**Figure 3:**
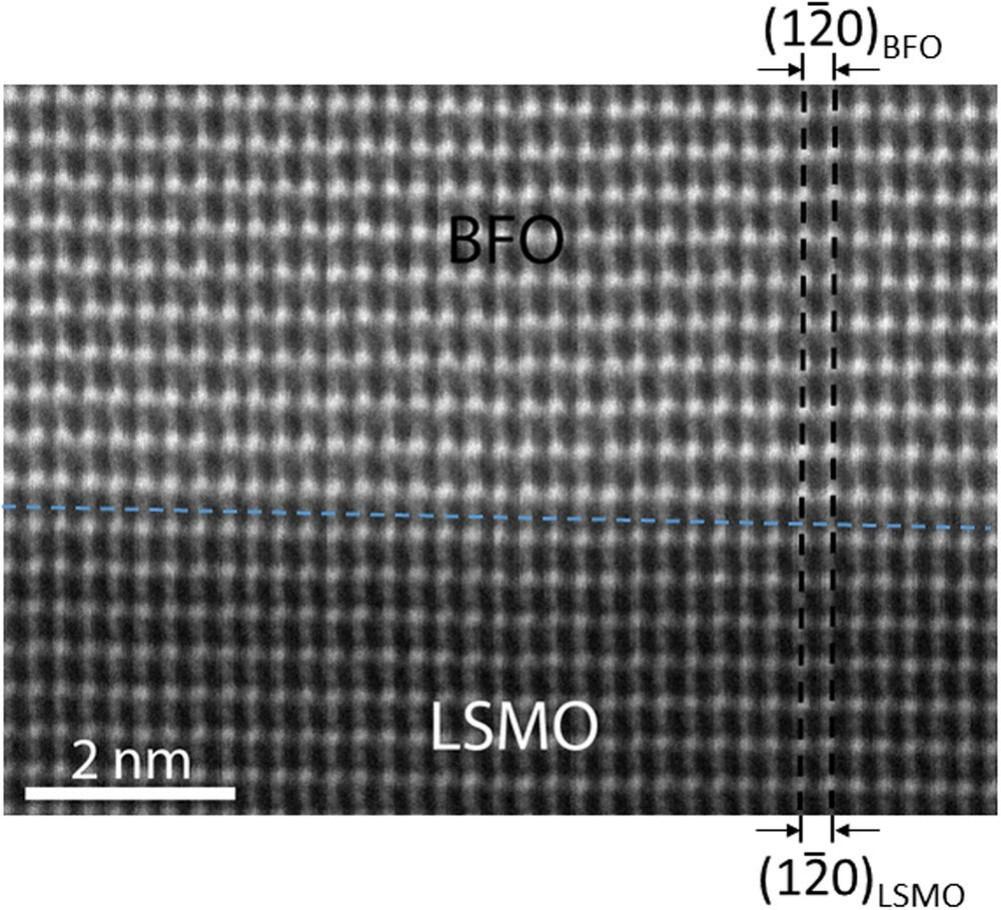
A cross-sectional HAADF-STEM image at the BFO/LSMO interface along [011]_STO_ zone axis. This demonstrates that the interface is atomistically coherent with no sign of lattice imperfection.

**Figure 4:**
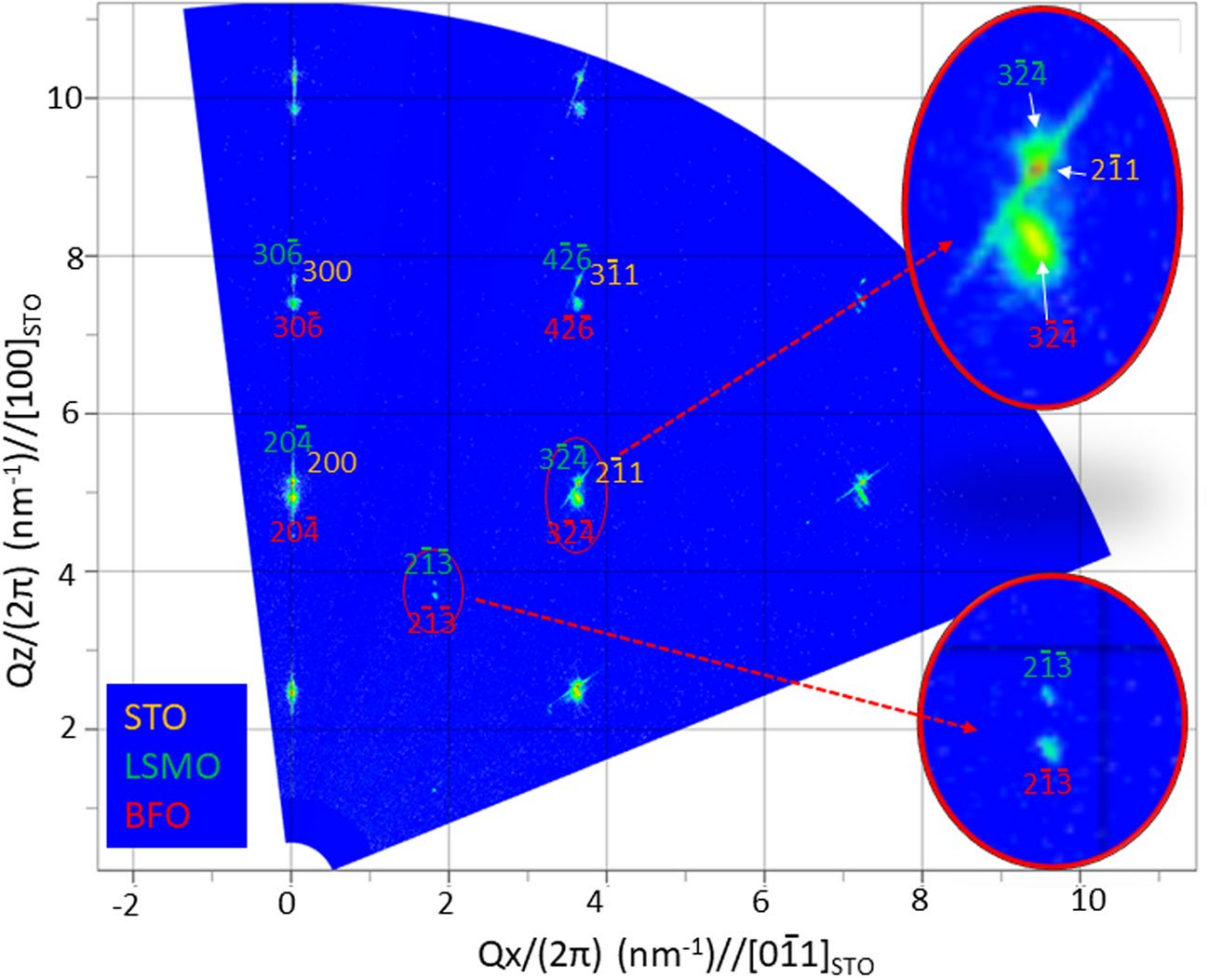
A XRSM data of the BFO film grown on LSMO/STO substrate, showing the existence of the rhombohedral signature Bragg’s reflections, i.e.,$\,2\overline{13}$ (see the bottom right inset) from BFO and LSMO. This also demonstrates the in-plane Bragg’s reflections of $3{\overline{24}}_{{\rm{LSMO}}}$, 2 $\bar{1}{1}_{{\rm{STO}}}$, and $3{\overline{24}}_{{\rm{BFO}}}$ are all lined up along Q_z_, i.e., out of plane, orientation.

In order to acquire more direct information about the structural detail about the BFO/LSMO interface, an atomic resolution high angle annular dark field (HAADF)-scanning TEM (STEM) image was acquired along [011]_STO_ zone axis, i.e., the same zone axis as in Figs 1 and 2, as shown in Fig. 3. The image readily shows that the ${(1\bar{2}0)}_{{\rm{BFO}}}$ lattice planes runs smoothly across the BFO/LSMO interface through ${(1\bar{2}0)}_{{\rm{LSMO}}}$ lattice plane with no sign of lattice imperfections such as dislocations or stacking faults that could be the sources of the strain relaxation at the interface. This directly demonstrates that the lattice strain caused by the lattice misfit between BFO and LSMO is exerted in BFO with no strain relaxation. Thus, the HAADF-STEM image is consistent with the strain contrasts shown in Fig. 1(a) and the NBED analysis result discussed with Fig. 2. Now that the lattice misfit is proved to be stored as the elastic energy, i.e., the lattice strain, within the BFO layer, it is worth focusing more on the details about the behavior of the lattice strain. In Fig. 4 is shown a wide range X-ray reciprocal space mapping (XRSM) that includes the Bragg’s reflections from BFO, LSMO and STO. Note that while a rhombohedral signature Bragg’s reflection, i.e., $2\overline{13}$, are clearly visible for both of BFO and LSMO (see the inset at the right-bottom for more details), this reflection does not exist for STO. This is consistent with the NBED result in Fig. 2 and further verifies the rhombohedral crystal structure identified for BFO. It is worth noting that the crystal structures together with ferroelectric polarization orientation within the BFO films grown on LSMO/STO was previously investigated by using quantitative aberration-corrected STEM technique which directly measures the locations of the Bi and Fe atoms in a HAADF-STEM image with pico-meter accuracy^31,32^. The BFO films were suggested to have either rhombohedral or tetragonal symmetries^31,32^. On the other hand, our result demonstrates that the rhombohedral symmetry of BFO can be unambiguously verified by using the conventional techniques of NBED and XRSM.

Another characteristic worthy of notice is that in-plane Bragg’s reflections for BFO, LSMO and STO are all lined up along out-of-plane orientation, indicating in-plane lattice planes, i.e., ${(1\bar{2}0)}_{{\rm{BFO}}}$, ${(1\bar{2}0)}_{{\rm{LSMO}}}$ and ${(0\bar{1}1)}_{{\rm{STO}}}$, have the same lattice spacing. This is consistent with the NBED results shown in Fig. 2. Since x-ray diffraction based techniques provide the superior accuracy to NBED in determining lattice spacings, the 30$\bar{6}$ and $4\overline{26}$ Bragg’s reflections are used to accurately derive the in-plane lattice spacings of $(1\bar{2}0)$ for the BFO and LSMO. As a result, they turn out 0.2759 for BFO and 0.2750 nm for LSMO, which match 0.2759 nm of $(0\bar{1}1)$ lattice plane distance in unstrained STO^30^. This confirms that the lattice spacings of BFO and LSMO are the same as that of STO along in-plane orientation. Note that the measured 0.2759 nm of ${(1\bar{2}0)}_{{\rm{BFO}}}$ is ~1.0% smaller than 0.27870 nm of the corresponding lattice plane spacing in *unstrained* bulk BFO^26^. This indicates compressive strain is applied in BFO layer along in-plane orientation. Now let us consider how the ~1.0% of the in-plane compressive strain affects the lattice plane along out-of-plane orientation by measuring the lattice plane distance along out-of-plane orientation. By using $30\bar{6}$ reflection of BFO, the lattice plane distance of $10\bar{2}$ is precisely calculated to be 0.4058 nm. This value is in good agreement with the out-of-plane lattice spacing, i.e., ~0.406 nm, measured for a BFO film grown on LSMO/STO by using quantitative STEM technique^33^. Note that this value is ~2.4% larger than 0.3961 nm of ($10\bar{2}$) lattice plane distance in unstrained bulk BFO^26^. The larger tensile strain value of ~2.4% along out-of-plane orientation than the ~1.0% of the compressive strain along in-plane orientation is considered to be associated with the fact that two-dimensional, i.e., biaxial, in-plane compressive strain effect shows up as one-dimensional, i.e, uniaxial, tensile stress along out-of-plane orientation. This is in agreement with the similar trend, i.e., the in-plane biaxial tensile strain causing a larger amount of uniaxial compressive strain along out-of-plane orientation, found in epitaxial BFO films previously^21,34^. Based on the results from NBED, HAADF-STEM and XRSM, it is concluded that the ~1.0% compressive strain exerted in BFO along in-plane orientation causes ~2.4% of tensile strain in BFO along out-of-plane orientation. Note that the current conclusion of rhombohedral crystal structure found within BFO film grown on LSMO/STO is in agreement with the previous works in that ~1.0% of the compressive strain imparted from LSMO/STO substrate is within the biaxial lattice strain range, i.e., between ~2.5% compressive and ~0.35% tensile strains, in which the rhombohedral crystal structure is found to be stable^23^. It is also noteworthy that the rhombohedral crystal structures found within the current BFO film could be slightly different in terms of the lattice parameter and rhombohedral distortion angle, i.e., *α* angle, from those found in other BFO films and bulk BFO. This is because the crystallographic details of the rhombohedal BFOs in terms of *α* angle, lattice parameter, and the locations of basis atoms depend on the characteristic lattice strain statuses, i.e., the amount and type of the particular lattice strain, induced by the particular lattice mismatch with the substrate material^23,24^.

**Figure 5:**
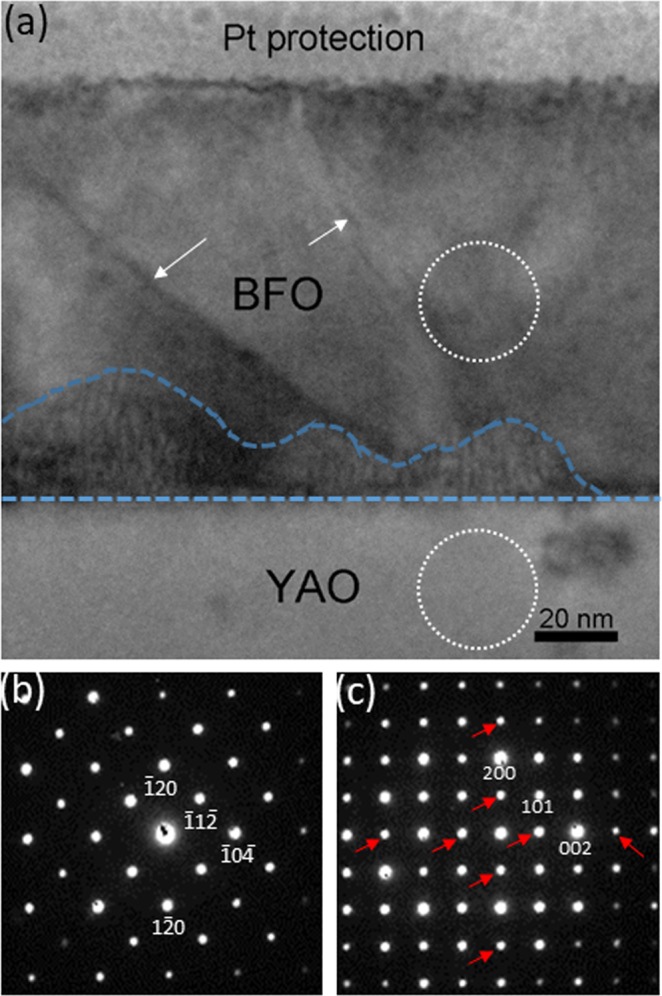
(**a**) A cross-sectional BF TEM image of BFO layer grown on YAO substrate along [010]_YAO_ zone axis. The contrasts due to low-angle grainboundaries are denoted with arrows. The area with high density of dislocations is indicated by blue lines. The NBED patterns from BFO and YAO are shown as (**b**) and (**c**), respectively. Note that the red arrows in (**c**) indicate the reflections caused by double diffraction.

Now let us turn our attention to the BFO film grown on YAO substrate. Figure 5(a) is a cross-sectional BF TEM image of a BFO layer grown on (100) YAO substrate, showing that a ~90 nm thick BFO layer grows on the YAO substrate. Note that the BFO layer shows the contrasts presumably associated with either low-angle grain boundaries or ferroelectric domain walls (denoted by white arrows)^35^, and the dislocations populated area (denoted by blue lines) at the BFO/YAO interface. The NBED patterns from BFO and YAO are shown in Fig. 5(b,c), respectively. Figure 5(c), i.e., for YAO, corresponds to that of [010]_YAO_ zone axis, whereas the four-fold symmetry showing up in Fig. 5(b), i.e., for BFO, could be interpreted for either [010] zone axis of cubicperovskite or [$42\bar{1}$] zone axis (in hexagonal notation) of rhombohedral as discussed previously^16,19,21^. Note that the red arrows in Fig. 5(c) indicate the reflections caused by double diffraction^28,29^. The characteristics of Bragg’s reflections in Fig. 5(b,c) are consistent with those in XRSM data (see Supplementary Figure S2), indicating that TEM data in Fig. 5 represent volume-averaged characteristics within the BFO film grown on YAO substrate.

**Figure 6:**
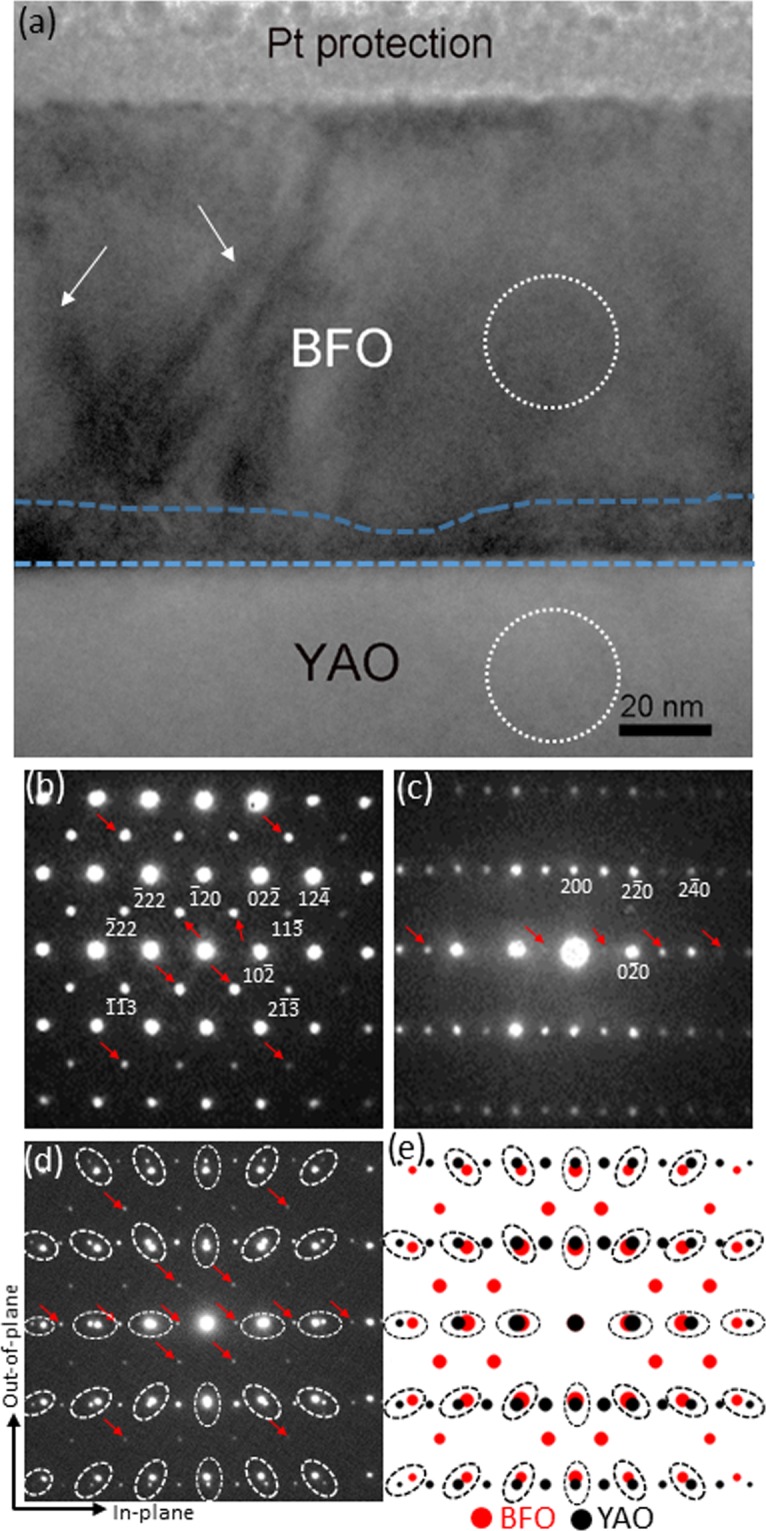
(**a**) A cross-sectional BF TEM image of BFO layer grown on YAO substrate along [001]_YAO_ zone axis. The NBED patterns from BFO, YAO, and the BFO/YAO interface are shown as (**b**–**d**) respectively. The structure factor calculation for the BFO/YAO interface is shown in (**e**) by using unstrained BFO and YAO materials. Note that the Bragg’s reflections denoted by red arrows are resulting from double diffraction.

In order to determine the crystal symmetry of BFO, another cross-sectional TEM sample is prepared along [001]_YAO_ zone axis as shown in Fig. 6. The BF image in Fig. 6(a) show highly similar characteristics with those found in Fig. 5(a) in terms of the contrasts attributable to either low-angle grain boundaries or ferroelectric domain walls (denoted by white arrows), and the area with high density of dislocations (denoted by blue lines) at the BFO/YAO interface. While the NBED pattern of YAO shown in Fig. 6(c) corresponds to [001]_YAO_ zone axis as expected, the NBED pattern of BFO in Fig. 6(b) clearly shows the rhombohedral signature Bragg’s reflections such as $2\overline{13}$, $11\bar{3}$, $\bar{2}13$ and $\overline{11}3$. These unambiguously identify the crystal symmetry of BFO as rhombohedral rather than cubicperovskite or others. Another NBED pattern is acquired at the BFO/YAO interface to investigate the lattice mismatch-induced strain status in BFO layer as shown in Fig. 6(d). Note that the reflections denoted with red arrows are resulting from double diffraction^28,29^. Unlike the NBED pattern at the BFO/LSMO in which the Bragg’s reflections from BFO and LSMO lined up along out-of-plane [(see Fig. 2(a)], Fig. 6(d) shows no sign of the particular orientation alignment in the Bragg’s reflections between BFO and YAO. In fact, if Fig. 6(d) is compared with the corresponding structure factor calculation using the crystallographic data of *unstrained* BFO^26^ and *unstrained* YAO^36^ materials [see Fig. 6(e)], it is readily noticed that the Bragg’s reflections split radially (with respect to the direct beam located at the center) between BFO and YAO in Fig. 6(e) (see the Bragg’s reflections encircled in black). This trend is identical to that in Fig. 6(d) (see those encircles in white), suggesting that the lattice mismatch between BFO and YAO induces *no lattice strain* in BFO, but is rather relaxed at the BFO/YAO interface. On the basis of the Figs 5(b,c), 6(b,c), the epitaxial relationship between BFO and YAO is summarized as follows:

3 $${[42\bar{1}]}_{{\rm{BFO}}}//{[010]}_{{\rm{YAO}}};{(\bar{1}20)}_{{\rm{BFO}}}//{(200)}_{{\rm{YAO}}}$$

4 $${[211]}_{{\rm{BFO}}}//{[001]}_{{\rm{YAO}}};{(\bar{1}20)}_{{\rm{BFO}}}//{(200)}_{{\rm{YAO}}}$$

**Figure 7:**
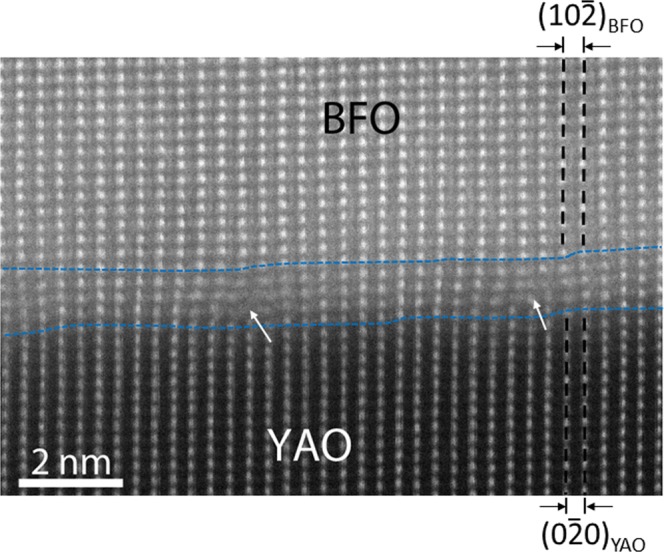
A cross-sectional HAADF-STEM image at the BFO/YAO interface along [010]_YAO_ zone axis. The dislocations (denoted by arrows) indicate that the lattice strain caused by the lattice mismatch between BFO and YAO is relaxed except for the heavily strained BFO area indicated by the blue lines.

In order to add more insight, an atomic resolution HAADF-STEM image was acquired at the BFO/YAO interface as shown in Fig. 7. Unlike the atomic resolution HAADF-STEM image at the BFO/LSMO interface in which the lattice planes of both BFO and LSMO are lined up with no sign of lattice imperfections (see Fig. 3), the BFO lattice plane along in-plane orientation, i.e., ${(10\bar{2})}_{{\rm{BFO}}}$, clearly show the existence of the dislocations as indicated by white arrows. This clearly indicates that ~6.8% of the lattice mismatch calculated between ${(10\bar{2})}_{{\rm{BFO}}}$ and ${(0\bar{2}0)}_{{\rm{YAO}}}$ is confirmed to be too large to be stored as the elastic energy, i.e., the lattice strain, within BFO layer except for a couple of nano-meter of the strained area denoted by the blue lines. On the other hand, ~1.0% of the lattice mismatch between ($1\bar{2}0$)_BFO_ and ($1\bar{2}0$)_LSMO_ along [011]_STO_ zone axis (see Fig. 3) turned out to induce the lattice strain with no sign of lattice imperfections. Thus, the current HAADF-STEM results in Figs 3 and 7 clearly demonstrate the importance of the atomistically coherent BFO/substrate interface for the lattice mismatch to be stored as the elastic energy, i.e., the lattice strain, in BFO film.

It is interesting to note that while some previous works have found the crystal structure of BFO grown on YAO as so called “supertetragonal” or “*T*-like” with the out-of-plane (*c*)/in-plane (*a*) lattice vector ratios ranging from ~1.23 to ~1.27^9,11^, the BFO grown on YAO in the current work turns out to have rhombohedral crystal structure. The discrepancy is considered to be associated with the different growth surfaces of the YAO substrates that provide different templates, i.e., different atomistic structures, for BFO to grow onto. For example, the in-plane lattice vectors of *b* and *c* for the (100) YAO used in the current study are ~0.7371 nm and ~0.5180 nm whereas the (110) YAO with a pseudocubic in-plane lattice parameter of 0.3704 nm (which is equivalent to the (010) YAO with in-plane lattice parameters of ~0.5330 nm and ~0.5180 nm in orthorhombic notation) was used in the previous works^9,11^. In fact, a very recent study showed that the crystal structure of the BFO grown on (100) YAO is rhombohedral^37^, which is in good agreement with the current study. This clearly demonstrates that: (1) the lattice strain status and the following crystal structure in BFO film are highly affected by the types of growth planes as well as the types of substrate materials; (2) the epitaxial relationship between BFO and substrate should be identified to quantitatively evaluate the misfit strain applied in BFO film.

## Summary

The crystal structures as well as lattice strain status were investigated for the BFO films grown on LSMO/STO and YAO substrates using ultra high vacuum r.f. magnetron sputtering. For the BFO film grown on LSMO/STO, the TEM and NBED results indicate that its crystal structure as rhombohedral. The epitaxial relationship identified by the NBED and precise lattice spacing measurement using the XRSM reveal that the BFO film is under ~1.0% of compressive lattice strain. The HAADF-STEM technique confirms the applied compressive lattice strain in BFO by directly showing the atomistically coherent BFO/LSMO interface. On the other hand, the crystal structure within the BFO film grown on YAO turns out rhombohedral with no sign of lattice strain from the NBED and structure factor calculation results although the lattice mismatch is estimated ~6.8% on the basis of the epitaxial relationship identified. HAADF-STEM technique clearly show the sign of the lattice strain relaxation, i.e., the dislocation formation at the BFO/YAO interface. This indicates that ~6.8% of lattice mismatch is too large to exert the corresponding amount of compressive lattice strain within the BFO film.

The current work demonstrates the highly synergetic combination effect of the TEM and XRSM techniques to precisely determine the crystal symmetry, epitaxial relationship, and lattice strain status within BFO films. The experimental results clearly show the importance of: (1) identifying the epitaxial relationship between the BFO film and the substrate material for the precise evaluation of the lattice mismatch, and (2) the atomistically coherent BFO/substrate interface for the lattice mismatch to exert the lattice strain.

## Methods

The BFO thin films were grown on a $(10\bar{2})$ LSMO buffered (100) STO and a (100) YAO substrates using ultra-high vacuum (<2 × 10^−6^ Pa) r.f. magnetron sputtering (ULVAC Co. Ltd.) at 550 °C. The detail about the deposition of the LSMO bottom electrode layer on STO substrate is given elsewhere^38^. The cross-sectional TEM samples were prepared by the focused ion beam technique, FEI Nova 600, with Ga ion beam. ~1 μm-thick Pt thin film was deposited on the surface of the sample to prevent the possible surface damage and re-deposition during the milling process. Then, the Ga ion beam energy gradually decreased from 30 to 1 keV to minimize ion beam induced damage. For atomic resolution HAADF- STEM analysis, a *Cs*-corrected TEM of JEOL JEM-ARM200F operated at 200 keV was used. For BF and NBED, a 200 keV JEOL JEM-2100F was used together with a Gatan Orius 833 CCD camera specifically designed with electron beam damage resistant scintillator. XRSM was performed using Rigaku SmartLab diffractometer with Cu*Kα* radiation.

## Supplementary information

Supplementary Figures
