# Supplementary material for: Short range biaxial strain relief mechanism within epitaxially grown BiFeO3
Source: Sci Rep. 2019 Apr 30;9:6715. doi: 10.1038/s41598-019-42998-x (PMC6491549; doi:10.1038/s41598-019-42998-x)
Supplement: Supplementary file 1 — Supplementary Figures [file 41598_2019_42998_MOESM1_ESM.pdf]

Supplementary Information

Short range biaxial strain relief mechanism within epitaxially grown $BiFeO_3$

In-Tae Bae[1,2]*, Shintaro Yasui[3], Tomohiro Ichinose[4], Mitsuru Itoh[3], Takahisa Shiraishi[5], Takanori Kiguchi[5] & Hiroshi Naganuma[4]

[1]Small Scale Systems Integration and Packaging Center, State University of New York at Binghamton, Binghamton, New York 13902, USA. [2]Department of Physics, State University of New York at Binghamton, New York, 13902, USA. [3]Laboratory for Materials and Structures, Tokyo Institute of Technology, 4259-J2-19, Nagatsuda-cho, Midori-ku, Yokohama, 226-8502, Japan. [4]Department of Applied Physics, Graduate School of Engineering, Tohoku University, Sendai 980-8579, Japan. [5]Institute for Materials Research, Tohoku University, Sendai 980-8579, Japan. *Correspondence and requests for materials should be addressed to I.-T.B. (e-mail: itbae@binghamton.edu)

Supplementary Figures

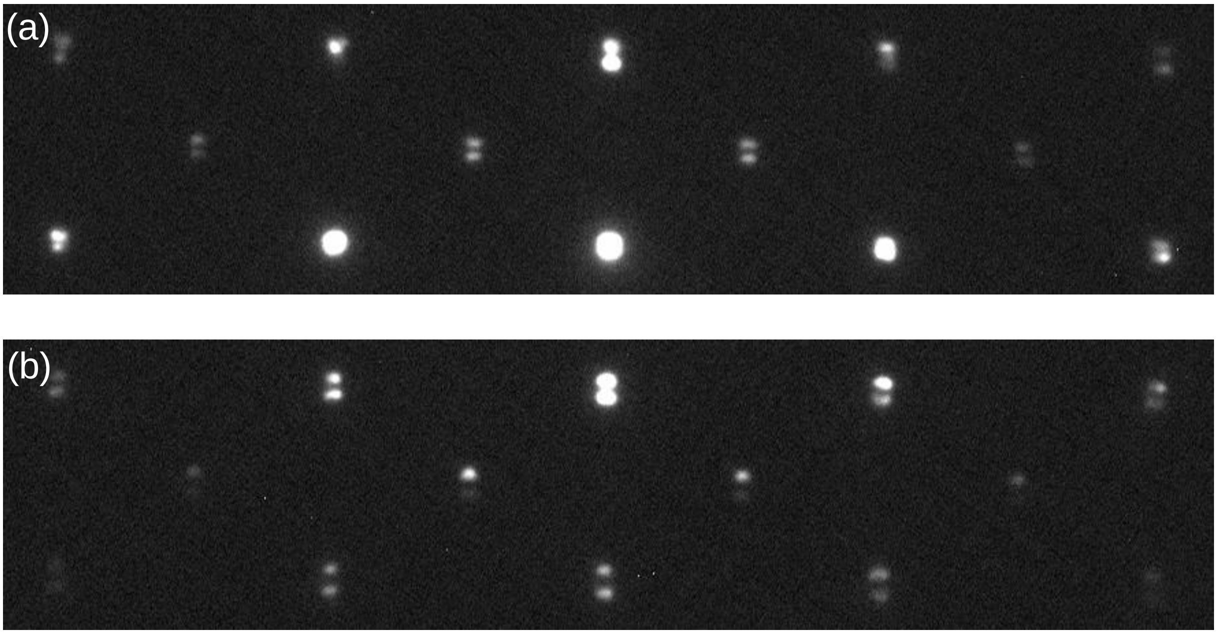

Supplementary Figure S1. Top (a) and bottom (b) areas denoted by rectangles in Fig. 2(a).

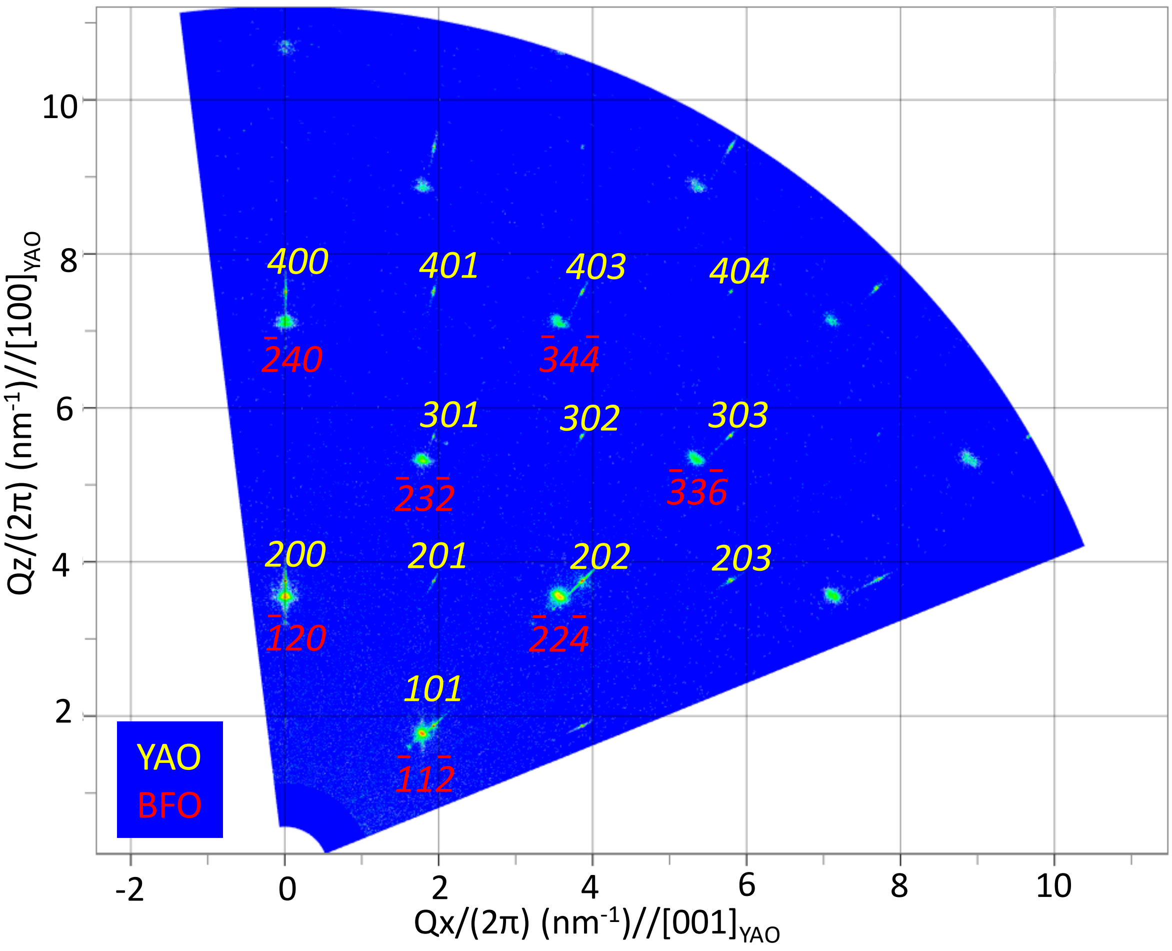

Supplementary Figure S2. XRSM data of the BFO film grown on YAO substrate.
